# Policy Considerations to Promote Equitable Cervical Cancer Screening and Treatment in Peru

**DOI:** 10.5334/aogh.3442

**Published:** 2021-11-24

**Authors:** Andrea Thoumi, Sarah J. Bond, Mary Elizabeth Dotson, Marlee Krieger, Patricia J. Garcia, Nirmala Ramanujam

**Affiliations:** 1 Health Equity, Duke-Margolis Center for Health Policy, 1201 Pennsylvania Ave NW, Washington, DC 20004, US; 2 Duke University, 100 Fuqua Drive, Durham, NC 27708, US Margolis Summer Intern, Duke-Margolis Center for Health Policy, US; 3 Center for Global Women’s Health Techologies, Duke University, Gross Hall, 140 Science Drive, Durham NC 27708, US; 4 Center for Global Women’s Health Technologies, Duke University, Gross Hall, 140 Science Drive, Durham NC 27708, US; 5 School of Public Health, Cayetano Heredia University, Lima-Peru, Av. Honorio Delgado 430, Lima 31 SMP, PE; 6 Robert W. Carr, Jr., Distinguished Professor of Biomedical Engineering, Center for Global Women’s Health Technologies, Duke University, Gross Hall, 140 Science Drive, Durham NC 27708, US

## Abstract

**Background:**

Cervical cancer is one of the leading causes of death among Peruvian women. Women seeking screening or treatment services experience delays in receiving screening results provided at community clinics or district hospitals, and lack sufficient resources to pay out-of-pocket to travel to the capital city of Lima for specialized treatment. Continued disparities in health outcomes and systemic barriers to accessing services suggest there are gaps between policy measures and implementation.

**Objectives:**

We aim to understand why national policies and clinical pathways that are aligned to global standards have been insufficient in improving cervical cancer screening and treatment in Peru, particularly among women who experience systemic exclusion from health services.

**Methods:**

We conducted a policy analysis based on a literature review (2005–2020), in Spanish and English, on PubMed, Global Health, Scopus, EconLit, Lilacs, and Scielo using a value-based care framework.

**Findings:**

The main barriers included unequal distribution of health infrastructure and health care workforce, and differences in access to health insurance. Additional barriers, including limited political will and support, limit efforts to prioritize the implementation of cervical cancer policies. We propose policy considersations in redesigning payment models, expanding healthcare workforce, generating costing and policy evidence, and reviewing policies for point-of-care technologies.

**Conclusions and Recommendations:**

The barriers identified in this literature review are applicable not only to cervical cancer care, but to primary health care in Peru. Systematic policy changes that address root causes of health inequities and are implemented at scale are needed to advance health reform efforts.

## Introduction

Cervical cancer is preventable and treatable in high-income countries; yet, it is a leading cause of death among women in Peru, an upper-middle income country in South America [1]. Incidence and mortality rates illustrate how systemic barriers to accessing screening and treatment and the interactions among other social and political determinants of health leave low-income, rural women at greater risk of morbidity and mortality. In Lima, the annual cervical cancer incidence rate is 19.2 new cases per 100,000 women, compared to the national rate of 32.7 new cases per 100,000 women [2]. Unlike high-income women who live in the capital city of Lima, low-income women residing in poorer peri-urban districts or in rural areas are more likely to die of cervical cancer [3]. Deep-rooted social and economic inequities lead to untimely access and poor-quality care, resulting in advanced-stage diagnoses and a striking health inequity: one in two women diagnosed with cervical cancer will die [4,5,6].

The typical clinical pathway for cervical cancer screening and treatment in Peru requires, at a minimum, three visits to the health facility for screening, colposcopy, and treatment [7]. Women seeking screening or treatment services experience delays in receiving screening results that are provided at community clinics or district hospitals, and lack sufficient resources to pay out-of-pocket to travel to the capital city of Lima for specialized treatment. It is common for a woman to wait a year between her first screening and the start of her treatment. Many women diagnosed with cervical cancer are indigenous and experience additional stigma, along with cultural and linguistic barriers, all of which exacerbate disparities in health outcomes [8,9,10,11,12]. In addition, hospitals are located at the edge of low-income communities without sufficient workforce or resources to provide high-quality care, and gaps in health insurance coverage result in unequal access to essential reproductive health services [13].

Global efforts to eliminate cervical cancer focus on vaccination and see-and-treat models that reduce the number of times a woman needs to visit a health facility to complete her screening and treatment. For example, since 2011, Peru implemented vaccination for human papilloma virus (HPV) for girls ages nine to 13. The country does not have a catch-up program to reach women from other age groups, a strategy that has shown increased benefits compared to a single age group vaccination strategy [14]. The single group vaccination leaves millions of Peruvian women unprotected from HPV, which is a well-established risk factor for cervical cancer. Additionally, although the national Peruvian demographic health survey reports that 61.8 percent of women 30 to 59 years old have had at least one Pap smear in the last three years, there are important differences and inequities between regions, and between rural and urban women [15]. Further, what this survey is not capturing is that Pap smear results are provided late, and women are lost to follow-up, so most of the cervical cancers in Peru are diagnosed in advance stage, reducing the likelihood of survival and contributing to the high mortality of this disease.

National policies to increase workforce capacity, expand access, and make care more affordable are aligned to cervical cancer guidelines with the World Health Organization (WHO) and Pan-American Health Organization (PAHO) [2,16,17,18]. Although overall mortality has decreased significantly over time, continued disparities in disease burden—including regional variation in mortality rates and access to timely, quality services—remain high [2,4,19]. Stemming from social and economic inequities, these disparities suggest policy measures should account for additional factors to overcome gaps between policy and implementation to improve health outcomes [5,6]. In this literature review, we aim to understand why national policies and clinical pathways that are aligned to global standards have been insufficient to improving cervical cancer screening and treatment in Peru, particularly among women who experience systemic exclusion from health services. Specifically, we conducted a policy analysis to identify enabling policy factors and barriers to cervical cancer screening and treatment in Peru to inform a national health policy agenda that prioritizes cervical cancer screening and treatment that is accessible among all women in Peru.

## Methods

We conducted a policy analysis based on a literature review using the Value-Based Care Framework developed by Duke University, which includes four domains: organizational competencies, care delivery innovation, financing and payment, and policies [20,21]. We augmented the literature review results with additional government and international organization resources [7,22,23,24,25,26].

### Policy Analysis

The Value-Based Care Framework was originally designed to assess accountable care reforms globally, and is described elsewhere [20,21,27]. A team from Duke University, in collaboration with global partners, developed the framework through stakeholder consultations with over 50 health policy and health leaders from around the world over the last five years. The stakeholder consultations and interviews informed in-depth case studies that captured key innovations in care, evaluation methods, patient outcomes, patient experience, and resource use and costs [21]. In addition, the team engaged with global leaders through in-country visits to understand capacity building needs, support implementation, and convene health stakeholders. Previous iterations of this framework have been applied to assess health system reform and policy changes in England, Germany, India, Mexico, Nepal, the Netherlands, Singapore, Spain, and Qatar [20,21,27,28]. Through this prior work, we have found that successful implementation of new care delivery models and technologies requires complementary changes in four domains: organizational competencies, care delivery innovations, financing and payment, and policies.

We adopted the four domains described in the Value-Based Care Framework as the conceptual framework for the policy analysis in this article. Domain 1 refers to organizational competencies in governance, health technology, finance, and care delivery among health care providers, policymakers, and purchasers needed to increase value. Domain 2 refers to care delivery innovations that enable health systems to deliver care that span medical and social needs. Domain 3 refers to health financing arrangements that link populations or episodes of care, not specific medical services, and measures to hold providers accountable to health outcomes. Domain 4 refers to policy measures that support population identification, performance measures, continuous improvement, care coordination, and alignment of financial and non-financial policies. Appendix 1 summarizes the review of the literature using these four domains.

### Literature Review

**Figure 1:**
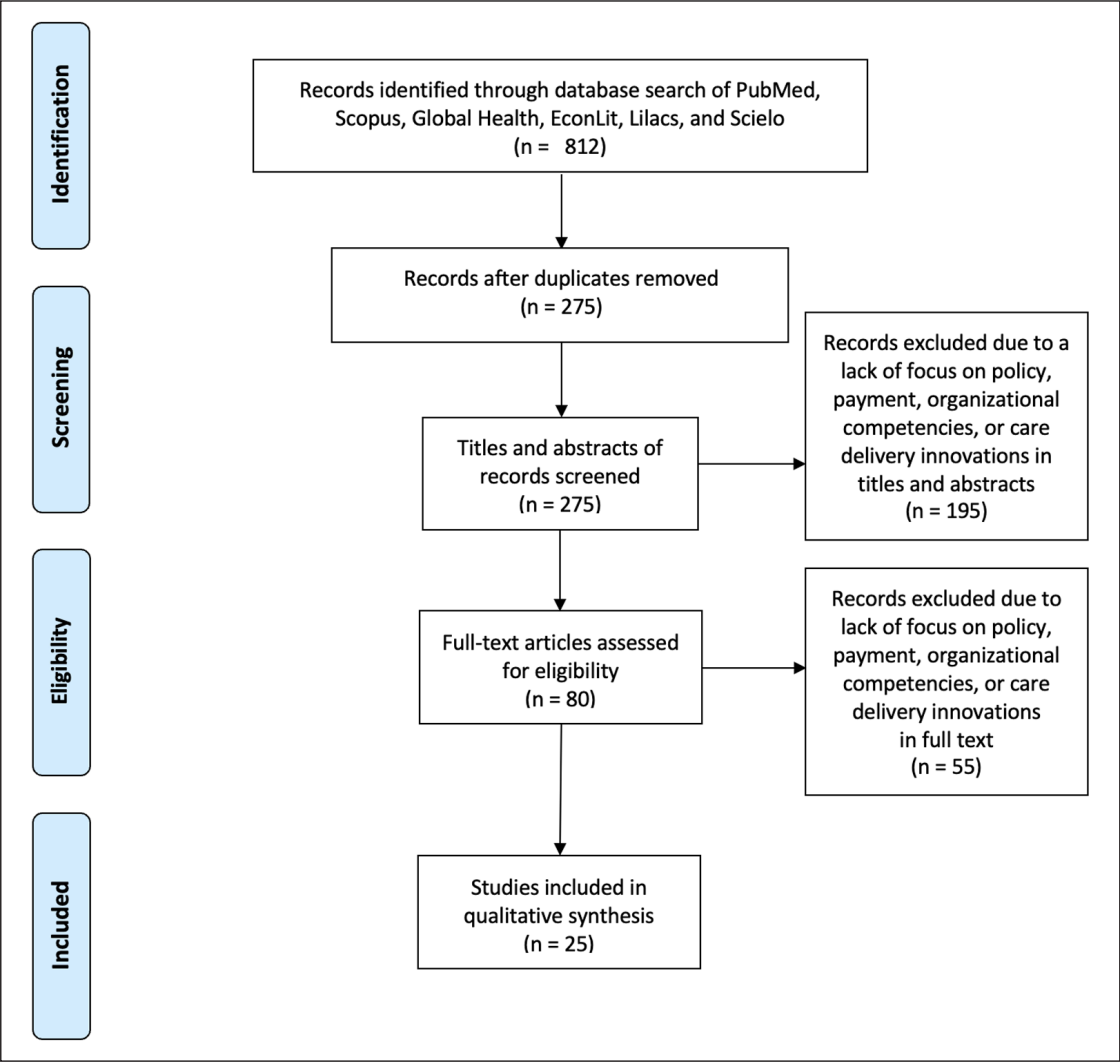
PRISMA Diagram of Literature Review.

We conducted a review of the literature between November 2019–March 2020 to identify articles for the review. ***Figure 1*** describes the results of the literature review using the PRISMA diagram. The team searched for articles published within the last 15 years (2005–2020), in Spanish or English, on PubMed, Global Health, Scopus, EconLit, Lilacs, and Scielo. The search strings included: “cervical cancer” + “Peru” + another key term. Key terms included “barriers”, “incentives,” “workforce,” “task shifting,” “policy,” “social determinants,” “innovation,” “performance measures,” “behaviors,” “technology,” “information technology,” and “evaluation.” The database search yielded 812 results. After duplicates were removed, 275 articles remained. To be included in our analysis, articles needed to address one or more of the four domains. In the first stage of review, one reviewer screened all titles and abstracts to ensure at least one domain was addressed; another reviewer screened 25 percent of excluded titles and abstracts. Based on screening of titles and abstracts, 80 articles were selected for full-text evaluation. During the full-text evaluation, we applied the same four domains as in the title/abstract screening to ensure at least one domain was addressed in detail in the text. Based on this full-text evaluation, 55 articles were excluded and 25 articles were included in the final policy analysis.

## Findings

We organize results from the policy analysis into four domains. In each of the domains we highlighted the key enabling factors and barriers for cervical cancer screening and treatment in Peru (***Table 1***).

**Table 1:** Key Enabling Factors & Barriers to Cervical Cancer Screening & Treatment.


	ENABLING FACTORS	BARRIERS	POTENTIAL SOLUTIONS

**Organizational Competencies**	The Peruvian government recognizes need to increase workforce and has identified in a recent policy how many health providers need to be trained in VIA, colposcopy, and cryotherapy [39]	Centralization of resources [1,11,13,29,30,32,40,41] Insufficient quality of testing and thus far, no implementation of HPV testing [16,31,33] Insufficient number & training of health providers [1,2,29,32,33,34,36,42] Loss of patients to follow-up [11,13,16,33,34,43,44]	Decentralize and monitor resource allocation Implement HPV testing (self-sampling) Train health providers considering capabilities needed at different levels (eg., at health centers vs. that at hospitals) Task-shift/task sharing to other train providers (eg., professional midwives) [22,26]

**Care Delivery Innovation**	Technological innovations [1,29,36,45,46,47]	No comprehensive system for evaluation of these innovations [17,33,34]	Create system for evaluation, accountability, and implementation Integrate emerging point of care technologies Awareness campaigns

**Financing & Payment**	National health insurance plans exist (SIS and EsSalud) [16]	Lack of awareness of public health insurance [38]	Payment reform which enables care outside hospitals Strengthen primary care and prevention actions

**Policies**	Alignment of Peruvian cervical cancer policy with guidelines of WHO & PAHO [2,16,17,18]	Failure to implement national policies [48]	Study policy challenges and evidence for in implementation Modify policies to address insufficient organizational competencies


### Organizational Competencies

Studies show that most health workers and infrastructure operate in Lima, the capital city of Peru. The unequal distribution of health workforce and infrastructure relative to need, limits access to preventive, diagnostic, and treatment services, and results in frequent health worker staffing shortages in laboratories and health facilities outside of the capital region [2,11,29,30,31,32]. Multiple studies cite inadequate training of specialists as a challenge [1,33,34]. One study posits that most providers are trained to provide care in hospitals, but do not learn techniques that are more commonly used in primary care settings, where providers have fewer resources at their disposal [1]. Insufficient training to collect sample and process the slides for Pap smears is closely linked to lower quality testing [2,11,33,34].

The Peruvian Ministry of Health (MINSA) recognized the need to improve organizational competencies, approving a National Plan for the Prevention and Control of Cervical Cancer 2017–2021, through a Ministerial Resolution in 2017. The National Plan identifies the total number of health providers currently trained in visual inspection with acetic acid (VIA), cryotherapy, and colposcopy and how many more providers should be trained by 2021. Furthermore, the Resolution recommends the doubling or tripling of health providers trained in VIA, cryotherapy, and colposcopy compared to 2016 rates. For example, as of 2016, there were 2,451 health providers trained in VIA, 349 physicians trained in cryotherapy, and an unknown number of health providers trained in colposcopy. The document recommended the following targets by 2021: 4,563 health providers trained in VIA, 1,399 physicians trained in cryotherapy, and 650 health providers trained in colposcopy [24]. However, actions to increase health workforce capacity were never implemented.

### Care Delivery Innovations

Studies focus on health technology innovations and mobile clinics to address barriers that deter screening and treatment efforts. These barriers include long travel times, waiting times, and stigma and discomfort surrounding pelvic examinations that lead to loss of follow-up [16,33,34]. Patients need to travel to the clinic multiple times for screening and treatment, and experience significant delays in receipt of Pap smear results from unresponsive health providers [16,35]. In addition, patient education regarding procedures that follow an abnormal result is limited. While mobile clinics can reduce these access barriers, mobile clinics can create additional barriers regarding workforce, maintenance, and recurrent supply costs, such as fuel, that make these models hard to sustain in Peru. In addition, the geographic terrain of the country in the Andes and Amazon regions hinders the implementation of mobile clinics.

Delivery innovations are shifting the location of screening to community- or home-based locations. A study reported that women found marketplace clinics to be easier to access and valued the fact that they could receive a Pap test near their home [35]. Another innovation, the use of a self-sampling molecular HPV test, aims to overcome the challenges of accessing a clinic and the gynecological examination. A study testing this methodology found that 74.2 percent of women were satisfied with the HPV self-sampling, women received their results using SMS messages to their cell phones, and 97 percent reported they would like to participate again in such a screening program [36]. Another community-based innovation is HOPE Peru, which trains women from communities where women at highest risk of cervical cancer reside to promote HPV self-testing, educate women about cervical cancer prevention, screening, and treatment, and refer HPV positive women to health centers.

Digital health tools and telemedicine have the potential to expand access to care in areas that lack adequate health system infrastructure, capacity, and human resources to provide prompt care. These solutions can support task-shifting activities or consultations with specialists to other health providers (e.g., midwives), an effective model for countries with limited resources [1,10,22,37]. Yet, reliance on cell phone technology will face additional challenges, such as connectivity to national mobile infrastructure and security of the devices in the health facilities. Furthermore, while these innovations are promising, the literature that we reviewed lacked comprehensive evaluations of the effectiveness of these novel care delivery approaches due to limited national or local capacity for monitoring and evaluation or rigorous quality control [17,33,34].

### Financing & Payment

Studies show differences in access to health insurance, and potential challenges in provider payment can result in variations in screening coverage for Pap smears and speed of receiving results. For example, sexually active women with public insurance through the Seguro Integral de Salud (SIS) are 1.27 times more likely to get a Pap smear than uninsured women whereas women with private insurance are 1.52 times more likely to get a Pap smear than uninsured women [16]. Furthermore, women who have private insurance typically receive faster diagnosis and treatment than those with public or no insurance [38]. One study suggests that the receipt of overtime bonuses by laboratory technicians can result in delays in sample collection and reporting results [11]. As a result, women wait to receive their test results, which can lead to loss of follow-up.

A key reason for these inequities in access and outcomes is the health financing structure in Peru. Peru has four health insurance schemes, including private health insurance, two national taxation-based plans, and a plan for all branches of the armed forces. The two national plans are SIS, which provides health insurance for low-income Peruvians, and the Seguro Social de Salud del Perú (EsSalud), which provides health insurance for workers with formal employment and their families. These programs enable some Peruvian women to afford cervical cancer screening; however, the funding is not equitably distributed across these four schemes. Nearly 70 percent of Peruvians depend on SIS, yet this scheme represents 30 percent of total health financing expenditures. In contrast, a 2017 WHO report estimated that private health insurance covers 30 percent of Peruvians, yet represents 70 percent of total health financing expenditures [23]. Another challenge is that some women who would qualify for SIS are not aware that SIS provides coverage for screening [38].

### Policies

The cervical cancer policy most frequently cited in the literature is Plan Esperanza, a cancer control plan that was launched in 2012. Its main objective was to increase access to cancer care services. To accomplish this goal, the policy was accompanied by an investment of $290 million [18]. With that funding, Plan Esperanza expanded access to care for low-income individuals by providing free cancer care coverage to individuals with SIS [38]. In fact, in 2014, Plan Esperanza provided cervical cancer screening with Pap to more than 2.3 million Peruvian women [2]. This policy also highlights the importance of geographic, economic, and cultural access to cancer care services [25]. Similar to many health policies that intend to improve access and reduce health inequities, this policy removed cost barriers once a woman reached the health system, but did not address the opportunity costs—including the time and cost to travel to the hospital or to Lima to receive care—that a woman incurs when she seeks screening or treatment [12].

As previously described, MINSA released National Plan for the Prevention and Control of Cervical Cancer 2017–2021, which was due to be implemented by 2021. However, this policy has not been implemented due to changes in governance and leadership structures within MINSA, and the political instability of the country [23]. The National Plan highlighted existing barriers to cervical cancer screening and treatment, including low coverage, delay in the return of test results, and poor follow-up for cases that required additional actions. It also described four key elements for implementation of the cervical cancer prevention and control strategy: 1) Improve the monitoring and evaluation system, 2) Improve coverage of testing by incorporating HPV testing, self-sampling, and provision of results via text messages, 3) Bring attention to pre-malignant cases to prevent cervical cancer, and 4) Improve care coordination for cervical cancer treatment, including task shifting and task sharing. Training was another core component of this policy. Specifically, the technical document emphasized the importance of rotations for health providers and training in the technique of VIA, cryotherapy, and HPV testing, and the creation of a monitoring system for training facilities [39]. As noted elsewhere, political will and support is a key factor to implementing screening and treatment programs at scale [10].

## Policy Recommendations

This literature review indicates the gap between policy and implementation is due, in part, to insufficient organizational competencies to implement models of care delivery that shift care from the hospital to the community, unequal distribution of health infrastructure and health care workforce [1,2,11,18,29,30,32], and differences in access to health insurance [38]. The centralization of resources to Lima continues to limit the ability of providers to a) implement national plans and b) diagnose and treat cervical cancer outside of the capital city [1,2,11,18,29,30,32]. In addition, the government has limited capability to conduct system evaluations of the effectiveness of new delivery approaches, which hinders country-level evidence generation [17,33,34]. Lastly, while the government has also expanded coverage for cervical cancer screening through public health insurance, limited awareness among women and out-of-pocket payments continue to hinder the uptake of coverage for reproductive health services [38,16].

In addition to these findings, limited political will, political instability, relatively weak monitoring, governance, and accountability mechanisms are other key barriers to implementing novel cervical cancer screening and treatment models in the country. For example, as noted in our findings, MINSA approved the National Plan for the Prevention and Control of Cervical Cancer 2017–2021, but the Plan was not implemented. The lack of transition from policy formulation to implementation for cervical cancer mirrors the trajectory of other policy reforms in Peru [5,49,50]. Furthermore, political instability and rapid changes in MINSA appointments have created a vaccum of political power to implement policies as intended and frequent changes to ministerial priorities. For example, over the last four years, there have been 11 Ministers of Health [51]. As a result, cervical cancer has not been given national priority, which has obstructed the national scale-up of a see-and-treat model that meets the needs of women to reduce the disease burden of cervical cancer. In addition, there has been limited buy-in to increase HPV self-sampling or moving colposcopies to primary care settings among key stakeholders, including gynecologists, due to financial implications of current financing models.

Efforts to shift national policies from theory to action will require a multi-pronged approach to overcome barriers in organizational competency, care delivery innovation, financing and payment, and policy. These efforts can build on the existing enabling factors we identified in the review, including recognition to increase workforce, development of new care delivery models, and increased coverage through SIS. Furthermore, proposed improvements to the health system’s financing and coverage design have the potential to support the health agenda that emerges after COVID-19. Possible areas for further policy dialogue include the following four areas and will require additional stakeholder engagement to identify priority ranking:

- Designing a payment package to enable providers to provide care outside of the hospital setting and move the services into primary care. This policy option will require reviewing existing provider payments for cervical cancer screening and treatment and development of a revised provider payment model to move care away from volume-based incentives toward outcome-based payment.
- Identifying a workforce to train and implement new community-based models in low-resource settings in the country. The government recognizes the lack of workforce, especially trained medical doctors, specialized gynecologists, and gynecologist oncologists. While task shifting is unlikely, task sharing among trained providers and with the provision of the supplies for screening and treatment of precancer lesions may be a potential alternative.
- Generating evidence, such as costing and policy analyses of existing policies, to inform the health policy agenda to advance community- and home-based primary care and essential services post-COVID-19.
- Reviewing policies related to the approval and distribution of new medical devices and new technologies for screening and treatment of precancer lesions and cancer.

These four potential policy solutions will need to extend beyond the scope of health policy reform to address systemic barriers to accessing care. For example, to increase the impact of national health insurance coverage, education campaigns can aim to increase awareness of public health insurance to ensure affordability of testing and treatment for all Peruvian women. In addition, MINSA can partner with the Ministry of Education to identify lessons from the roll-out of tablets for school-aged children during COVID-19 to understand barriers and solutions to implementing technology-based solutions that will require the distribution of new technology with connectivity to the mobile infrastructure. This issue will be especially salient for the implementation of self-testing solutions or devices that require use of a tablet or smartphone on a national level. Lastly, the frequent change of health policy leadership may continue to hinder the transition from policy formulation to implementation.

Limitations of this literature review are the following. While we included key government and WHO documents, the review only included peer-reviewed findings. This excludes insights that may have been reported elsewhere. Another limitation is that the student team who did the initial article screening did not adopt a conventional double screening process due to limited Spanish language proficiency on the team. Lastly, the analysis was conducted four months after the literature search was completed.

## Conclusion

Inequities in resource allocation and access to services are the root causes of Peruvian women lacking timely access and high-quality care for reproductive health. Efforts to increase access to cervical cancer screening and treatment need to provide women culturally accessible strategies that focus on their experiences, the quality of the care they receive, and their health outcomes. The WHO has established clear metrics to reduce the inequities in cervical cancer mortality by 30 percent in the next decade through increased vaccination, screening, and treatment [52]. Increased use of community-based models that promote task-shifting coupled with medical innovations in HPV testing, artificial intelligence, imaging, and thermal ablation can help achieve this goal. In addition, policy and payment changes will be needed to address systemic root causes to ensure equitable access to high-quality prevention, screening, and treatment services for cervical cancer among all women in Peru and around the world.

## Additional File

The additional file for this article can be found as follows:

10.5334/aogh.3442.s1 Appendix 1. Data Collection using the Value-Based Care Framework.
