# Supplementary material for: Policy Considerations to Promote Equitable Cervical Cancer Screening and Treatment in Peru
Source: Ann Glob Health. 2021 Nov 24;87(1):116. doi: 10.5334/aogh.3442 (PMC8622178; doi:10.5334/aogh.3442)
Supplement: Appendix 1. — Data Collection using the Value-Based Care Framework. [file agh-87-1-3442-s1.pdf]

Appendix

Appendix 1: Data Collection using the Value-Based Care Framework

Author	*Study Purpose*	*Policies*	*Financing & Payment*	*Organizational Competencies*	*Care Delivery Innovations*
Aguilar et al. (2016)	*To identify barriers for cervical cancer control*	*Plan Esperanza was launched in 2012, which focuses on the promotion of cancer prevention and health worker training programs.*	*Budget by Results is part of the Comprehensive National Cancer Control Plan.*	*Loss of follow-up and insufficient number of specialists in rural areas are barriers to cervical cancer screening and treatment.*	*Mass HPV vaccination is suitable for the Peruvian population.*
Albújar-Baca (2010)	*To assess regional screening coverage for cervical cancer*	*This paper cites lack of a unified cancer control plan as a barrier to effective screening and treatment.*	*n/a*	*Low level of regional screening— should be testing 70–80% of the population, but only 9% are being tested; insufficient # of cytotechnologists*	*n/a*
Almonte et al. (2015)	*To compare cervical cancer screening tests*	*n/a*	*n/a*	*Key barriers include insufficient number of specialists to screen patients and poor collection and labeling of samples.*	*Study found that HPV testing performed best of the four cervical cancer screening tests. One barrier related to care delivery innovation was lack of quality control mechanisms.*

Author	*Study Purpose*	*Policies*	*Financing & Payment*	*Organizational Competencies*	*Care Delivery Innovations*
Anticona et al. (2015)	*To identify barriers for cervical cancer control, based on the perspectives of doctors in rural areas*	*n/a*	*n/a*	*Key barriers include shortage of specialists, insufficient diagnostic equipment, and patient-related barriers inhibiting follow-up.*	*Doctors working in rural areas suggested that increasing the amount of point-of-care diagnostic tools and the use of mHealth apps would improve cervical cancer screening and treatment.*
Barrionuevo-Rosas et al. (2013)	*To determine impact of health insurance type on likelihood for screening*	*The WHO recommends that women get tested for cervical cancer every 3 years if they are over the age of 25, with increased emphasis on women 30–49 years old.*	*An enabling factor for cervical cancer screening and treatment is the existence of public health insurance programs. Notably, even with public health insurance, co-payments are required for screening and treatment.*	*Key barriers include low coverage for pap smears, insufficient quality in pap processing, and a low rate of follow-up and treatment of women with abnormal cytology.*	*n/a*
Bartolini et al. (2010)	*To understand the context surrounding cervical cancer in order to inform HPV vaccine introduction*	*Policymakers expressed general support for HPV vaccine introduction via the National Immunization Strategy.*	*n/a*	*Vaccine coverage is generally high in Peru, at least 93% (depending on the vaccine) in children less than one year old as of December 2006. Notably, health workers need additional training to prevent incorrect vaccination administration.*	*Peru has a comprehensive system for monitoring, investigating, and reporting adverse events attributed to vaccination but it is difficult to track whether individuals have received all doses of multiple-dose vaccines like HPV*

Author	*Study Purpose*	*Policies*	*Financing & Payment*	*Organizational Competencies*	*Care Delivery Innovations*
Bayer et al. (2011)	*To determine missed opportunities for health education*	*Cervical cancer screening has been a priority among the Peruvian Ministry of Health for over ten years, with recommendations for Pap smear screenings every 3 years for women between 30 and 49 years of age, and starting at age 25 if possible.*	*n/a*	*In terms of health education related to cervical cancer, only one in five provider visits observed included some type of health education on the disease and its prevention. Additionally, follow-up was found to be limited with 20% of women receiving no follow-up and 50% of women receiving recommendation to get follow-up at "some point in the future."*	*n/a*
Colantonio et al. (2009)	*To assess cost-effectiveness of HPV vaccine introduction*	*n/a*	*n/a*	*n/a*	*Universal mass HPV vaccination is a cost-effective strategy for reducing cervical cancer prevalence.*
Ferris et al. (2019)	*To analyze Peruvian women's perspectives on new financing strategies designed to improve rate of follow-up for cervical cancer cytology results*	*Ministry of Health provides free screening to many women.*	*A reimbursement incentive program designed to improve follow-up of cervical cytology test results was acceptable to most Peruvian women.*	*36% of women had previous problems receiving cervical cytology results, resulting in patient distrust of cervical cancer screening programs, ambivalence in having additional cervical cytology tests, and reluctance to exert an effort to obtain results in the future.*	*79% of women in study own a cell phone—Implementation of widespread patient notification of lab results by phone should be seriously considered by all cervical cancer screening facilities in Peru, including the Ministry of Health.*

Author	*Study Purpose*	*Policies*	*Financing & Payment*	*Organizational Competencies*	*Care Delivery Innovations*
Ferris et al. (2015)	*To determine the impact of a mobile medical clinic on cervical cancer screening*	*n/a*	*n/a*	*n/a*	*Many poor indigenous women living in isolated regions are unable to travel to distant healthcare facilities. Using a novel mobile clinic model, the "Dia del Mercado Project" successfully reduced barriers to cervical cancer screening by using local marketplaces.*
Goldie et al. (2005)	*To determine cost-effectiveness of cervical cancer screening strategies*	*n/a*	*The most cost-effective strategies were those that required the fewest visits, resulting in improved follow-up testing and treatment.*	*The highest cost for women is not directly related to the medical system, but to women's travel and time.*	*n/a*
Gutiérrez-Aguado et al. (2011)	*To determine cost-effectiveness of HPV vaccination*	*n/a*	*n/a*	*Insufficient national coverage with Pap smear*	*The HPV vaccine is cost-effective, compared to no vaccine.*
Jeronimo et al. (2005)	*To assess visual inspection with acetic acid (VIA) as a means for cervical cancer screening*	*n/a*	*n/a*	*Shortage of specialists results in long wait time to receive results of Pap screening (1–3 months). Few health centers have tools to treat preinvasive lesions.*	*Visual inspection with acetic acid is more likely to result in earlier diagnosis, follow-up, and treatment than Pap smear.*
Johnson et al. (2018)	*To determine enabling*	*Plan Esperanza*	*"Those with private*	*Barriers include*	*n/a*

Author	Study Purpose	Policies	Financing & Payment	Organizational Competencies	Care Delivery Innovations
	factors and barriers to cervical cancer treatment	*allowed low-income Peruvians to pay for care.*	*insurance usually received faster diagnosis and treatment and experienced fewer institutional barriers than participants with public or no insurance."*	*limited resources at facilities, lack of awareness about SIS, long wait times, and poor coordination of care.*	
Luciani et al. (2008)	*To determine effectiveness of crytotherapy treatment*	*n/a*	*n/a*	*"25% of women with an abnormal Pap test actually received follow-up for diagnosis and treatment, and this was attributed to poor infrastructure, problems with communication of results, multiple visits required for diagnosis and treatment, and travel-related reasons"*	*Cryotherapy is an effective treatment for cervical precancerous lesions; it can easily be administered by general practitioners in primary care settings following visual inspection screening.*
Luciani et al. (2011)	*To assess effectiveness of visual inspection with acetic acid (VIA) as a means for cervical cancer screening*	*n/a*	*n/a*	*Key barriers include poor-quality testing, low coverage, and difficulty in diagnosis.*	*Visual inspection with acetic acid can lower the population risk for cervical cancer.*
Luque et al. (2016)	*To assess perspectives of community outreach program*	*"Peru's cervical cancer screening guidelines are targeted at women between 30 and 50 years and include annual Pap tests, VIA every 2 years, and the HPV DNA test every 5 years"*	*n/a*	*Health centers lacked resources and women had difficulty accessing treatment due to centralization of resources in Lima.*	*CerviCusco provided screening outreach campaigns to increase knowledge surrounding cervical cancer screening and treatment.*

Author	Study Purpose	Policies	Financing & Payment	Organizational Competencies	Care Delivery Innovations
Mendoza-Cervantes et al. (2017)	To describe a network designed to discuss challenges & support implementation of Plan Esperanza	Plan Esperanza emphasized importance of decentralization, highlighting the critical role of advocacy and education at the local level. Minister of Health, Patricia Garcia, stressed need to improve coordination between national and regional governments. Paper notes that cancer control plans lack continuity.	n/a	Unequal distribution of medical expertise. Some providers lack training in cervical cancer prevention.	n/a
Morán et al. (2017)	To determine perspectives on self-administered HPV tests	n/a	n/a	n/a	74.2% of the users felt, at least, satisfied with the HOPE program. 68% of women prefer to take the self-administered HPV test.
Mueller et al. (2017)	To compare Pocket Colposcope with standard of care	n/a	n/a	n/a	See below.
Mueller et al. (2018)	To compare Pocket Colposcope with standard of care	n/a	n/a	n/a	"The Pocket colposcope performed similarly to a standard-of-care colposcope when used to identify pre-cancerous and cancerous lesions using acetic acid and Lugol's iodine during colposcopy exams in Peru"
Paul et al. (2013)	To examine enabling	n/a	n/a	Institutional factors,	Use of visual acetic

Author	*Study Purpose*	*Policies*	*Financing & Payment*	*Organizational Competencies*	*Care Delivery Innovations*
	factors and barriers to screen-and-treat programs			*such as long wait times & timing of services; long lines to receive services & inconvenience of services only being offered in the mornings; lack of trust, particularly regarding cleanliness of device*	*acid inspection is an effective approach for cervical cancer screening. Activities that can help ensure successful program include mobilizing and educating communities, organizing services to meet women's schedules and needs, and strengthening systems to track clients for follow up.*
Paz-Soldan et al. (2012)	*To identify barriers to screening and treatment of cervical cancer*	*National Cancer Institute's 2008 Manual of Standards and Procedures for Cervical Cancer Prevention mandates that all sexually active women aged 30–49 or women with at least one risk factor should have a Pap smear every three years. Notably, problems with implementation prevent this from being a reality.*	*Perverse incentives increase the lag time between sample collection and return of results.*	*Widespread lack of resources and centralization of treatment and diagnostic tools in Lima. Patients are lost to follow-up. Increased specialist training must be provided.*	*n/a*
Ministerio de Salud (2017)	*To establish the technical processes for early*	*This policy describes screening and*	*n/a*	*n/a*	*n/a*

Author	*Study Purpose*	*Policies*	*Financing & Payment*	*Organizational Competencies*	*Care Delivery Innovations*
	detection and treatment of cervical cancer	*treatment techniques, and establishes that all women aged 25–64 are eligible for a free cervical cancer screening, financed by "Prevencion y Control del Cancer."*			
Zafra-Tanaka et al. (2018)	*To determine perceptions of physicians regarding their own competence in diagnosing cervical cancer*	*WHO and PAHO guidelines recommend visual inspection with acetic acid because it's less expensive, non-invasive, and requires little equipment and infrastructure*	*n/a*	*Six out of ten recently graduated physicians perceived themselves as adequately competent to perform VIA or VILI, likely because training is centered in hospitals, where pap smears are more common.*	*Physicians interviewed suggested using smartphones to consult with experts when uncertain about a diagnosis using visual inspection with acetic acid.*
